# Primary Rifampicin Mono-Resistant Extrapulmonary Tuberculosis of the Scapula With Cold Abscesses: A Report of a Rare Case

**DOI:** 10.7759/cureus.58598

**Published:** 2024-04-19

**Authors:** Sankalp Yadav

**Affiliations:** 1 Medicine, Shri Madan Lal Khurana Chest Clinic, New Delhi, IND

**Keywords:** rifampicin mono-resistance tuberculosis, cbnaat/ xpert/ rif assay, mtb (mycobacterium tuberculosis), tuberculosis, scapula

## Abstract

Extrapulmonary tuberculosis is rare. Tuberculous involvement of the scapula is an infrequently reported entity. Such cases are exceptionally rare, as there is no documented case of an isolated primary rifampicin mono-resistant extrapulmonary tuberculosis of the scapula with cold abscesses in the medical literature. This case report features a 25-year-old Indian male patient whose main complaint was a painful swelling with a discharging sinus in his left shoulder that limited his range of motion. A thorough blood workup, clinical assessment, and scans led to a definitive diagnosis. The patient was commenced on antituberculous therapy.

## Introduction

Tuberculosis is a major public health concern in endemic nations [1]. *Mycobacterium tuberculosis*, an acid-fast bacillus that belongs to the Mycobacteriaceae family, is the underlying cause of the disease [2]. According to data from the most recent reports from India, 119,000 patients (93,000-145,000) on average were projected to be multidrug-resistant or rifampicin mono-resistant in 2021. Furthermore, compared to 2021, there was a 32% rise in the number of multidrug-resistant and rifampicin mono-resistant cases detected in 2022 [3].

Nearly 15% of extrapulmonary tuberculosis cases and 1% of all tuberculosis cases are skeletal tuberculosis cases, with the spine being impacted in 50% of the cases [4]. It is uncommon to get isolated tuberculous osteomyelitis in flat bones like the scapula. The same is evident from a very few reports on tuberculosis affecting the scapula [5]. However, none of these are of the drug-resistant type. Herein, a case of primary rifampicin-mono-resistant extrapulmonary tuberculosis of the left scapula with cold abscesses is presented.

## Case presentation

A 25-year-old non-diabetic Indian male belonging to a middle-income family reported to the outpatient department as a referral case. He presented with complaints of pain, swelling, and purulent discharge from his left shoulder. The swelling had an insidious onset over the last four months and was associated with pain and a yellow-colored, non-foul-smelling discharge for the past 15 days.

He was a stationery shop owner with no history of tuberculosis among his contacts. However, he had a history of a road-traffic accident 1.5 years ago where he sustained a fracture of the left clavicle. He was treated for the same at a local orthopedic clinic, on which no details are available. There was no history of constitutional signs of tuberculosis, and there was no history of substance abuse or stays at crowded places, such as prisons and refugee shelters.

**Figure 1:**
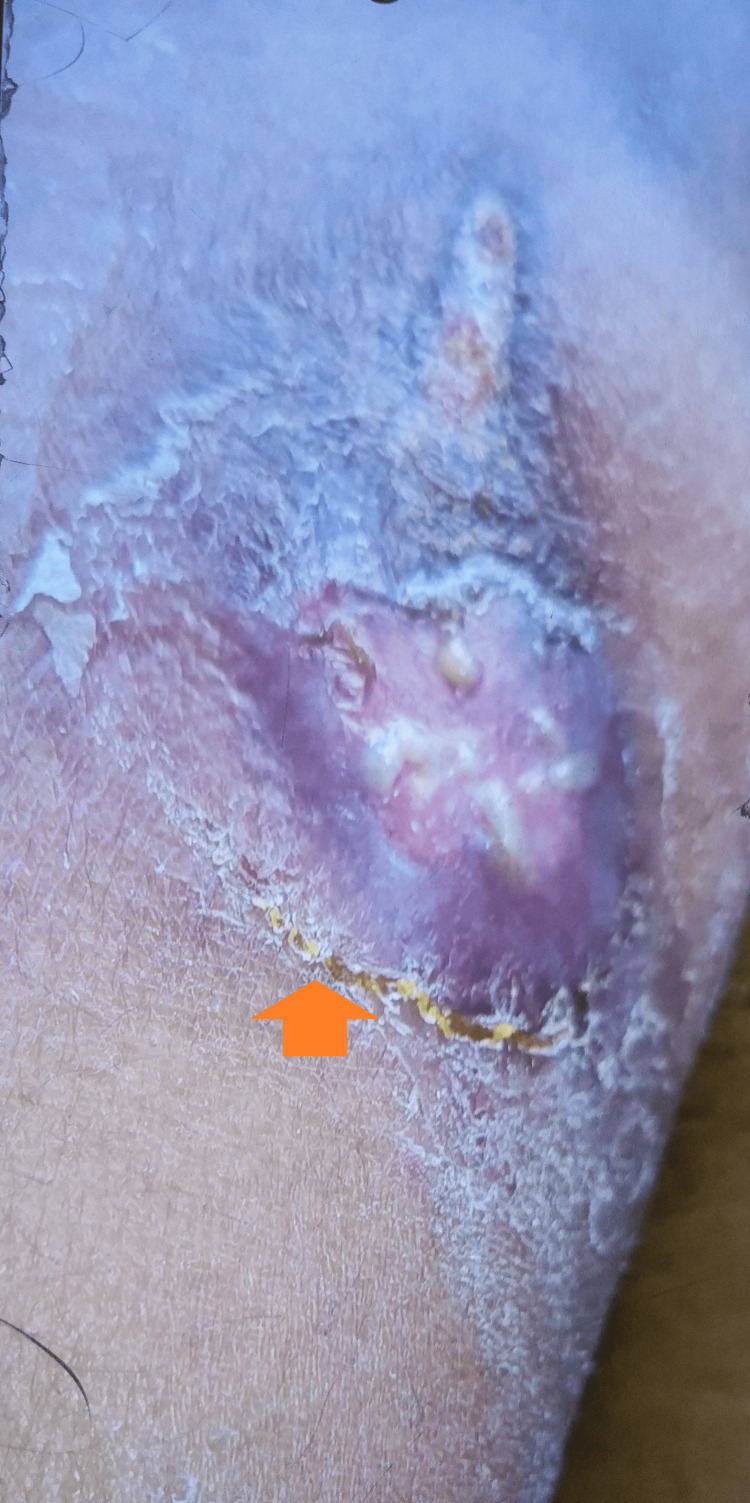
Gross image at presentation showing the wound with a purulent discharge

A general examination was suggestive of a young man with a weight of 78.9 kg and afebrile to touch. He had blood pressure of 120/80 mm of Hg, pulse of 73 per minute, and peripheral oxygen saturation at room air of 98%. His systemic examination was not suggestive of any disease. The local examination was remarkable for a swollen left shoulder with a discharging wound from the proximal arm measuring about 9 x 5 cm with clear edges and purulent discharge coming from it (Figure 1).

**Figure 2:**
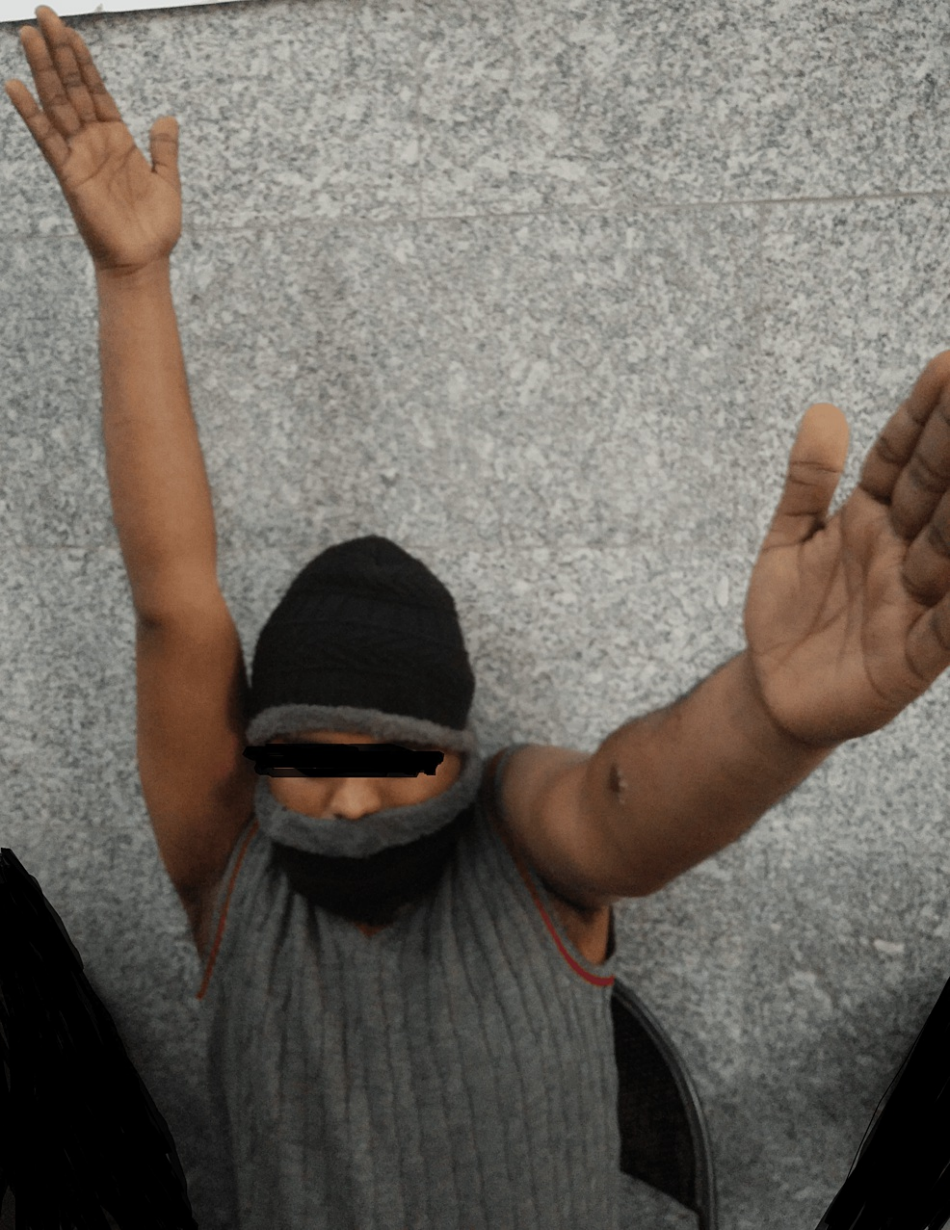
Restricted range of motion on the left side

Further, there was no clubbing, cyanosis, icterus, or pedal edema, but there was left axillary lymphadenopathy. Overhead abduction was restricted on the left side (Figure 2).

**Figure 3:**
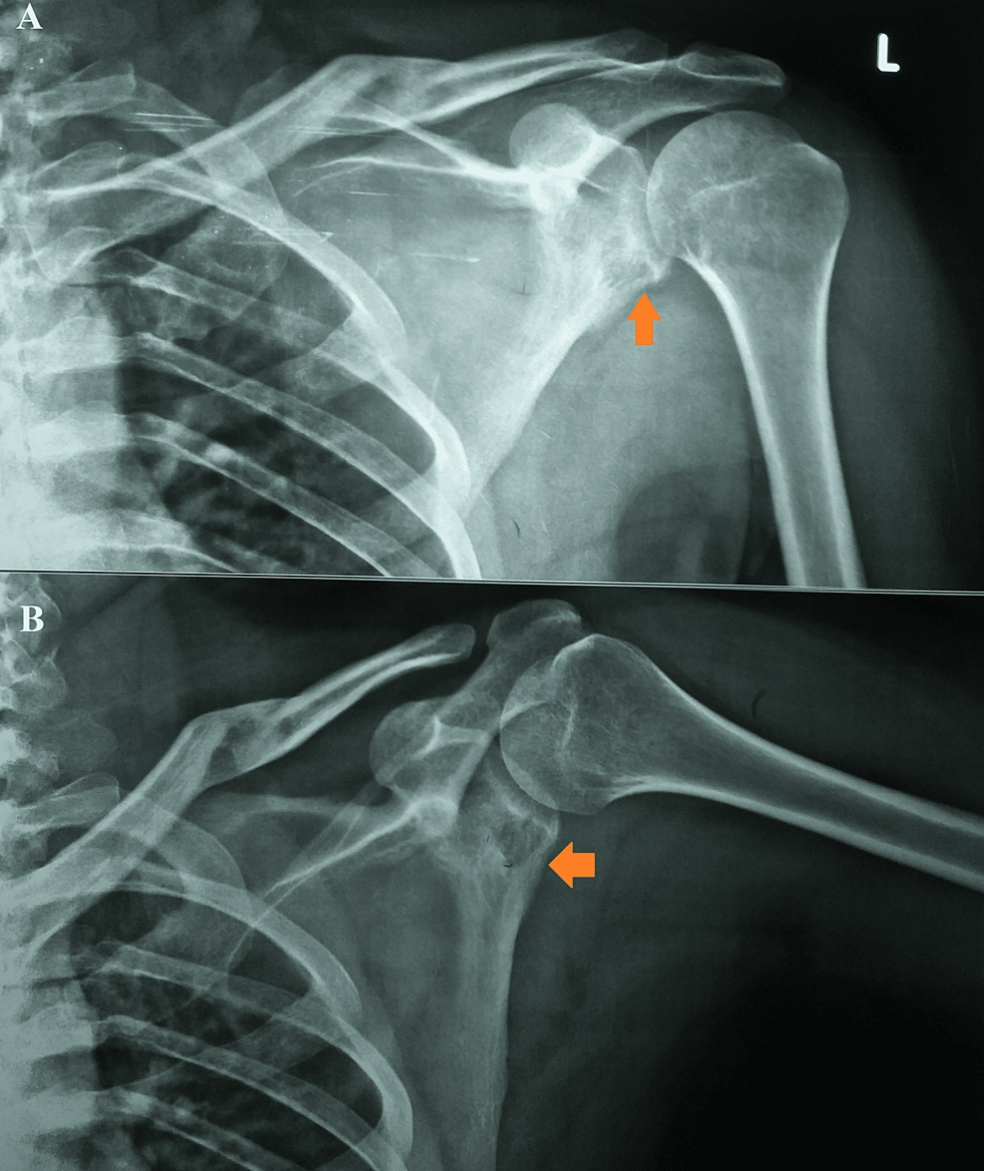
A plain radiograph of the left shoulder joint suggestive of a sclerotic lesion in the neck of the left scapula. A,B: anteroposterior view

A plain radiograph of the left shoulder joint was suggestive of a sclerotic lesion in the neck of the left scapula, and an old fractured left clavicle with a malunion was seen (Figure 3).

A diagnostic laboratory workup, presuming he was a case of pyogenic abscess with differentials including tuberculous osteomyelitis, bone tumor, and fungal osteomyelitis, was remarkable for a raised erythrocyte sedimentation rate (52 mm in the first hour), a raised C-reactive protein (36.40 mg/L), and a positive Mantoux test (20 x 20 mm induration). Additionally, his induced sputum examination and cartridge-based nucleic acid amplification of the same were negative. His HIV (I and II) and hepatitis panels were also negative. His chest X-ray was normal.

**Figure 4:**
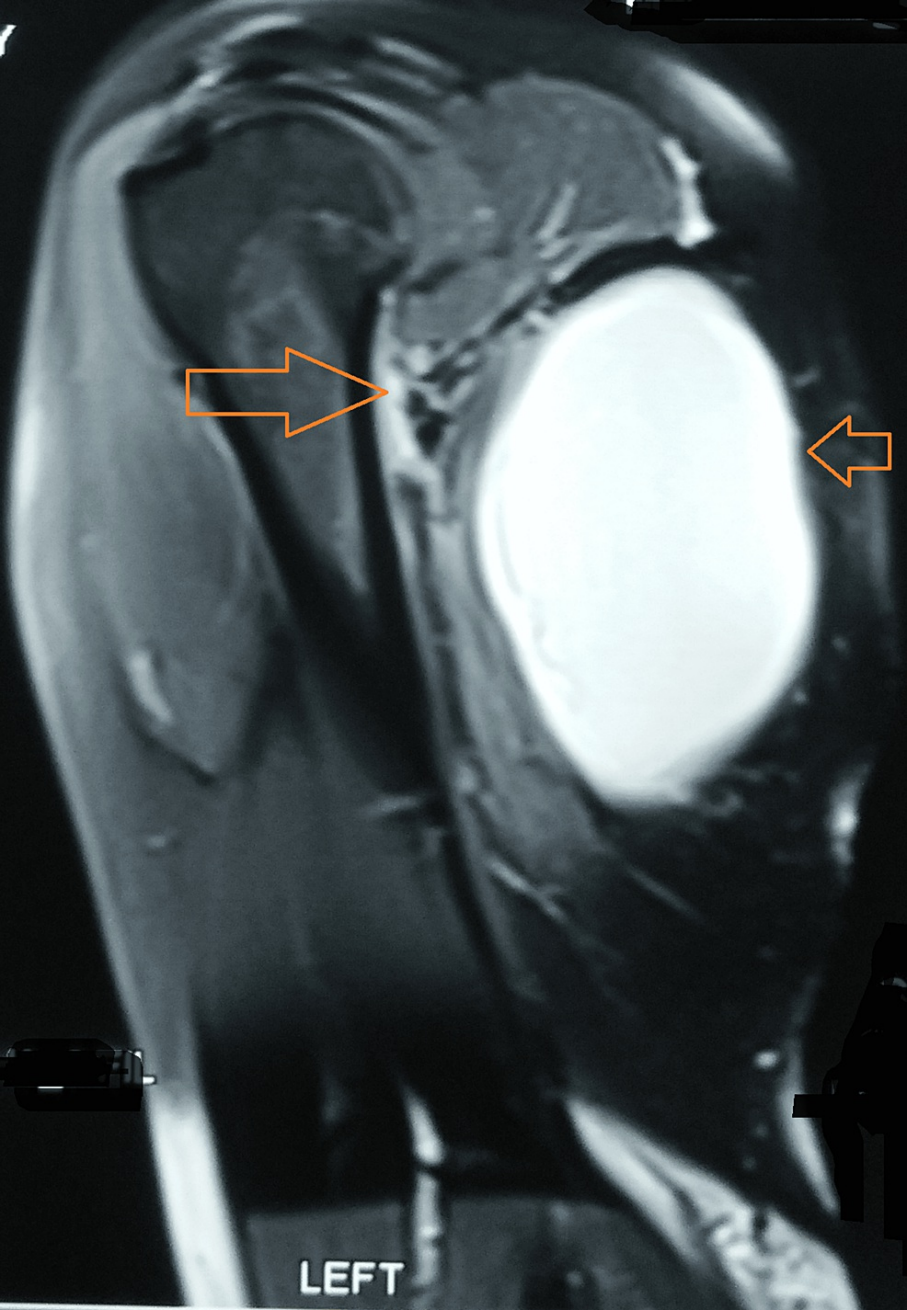
MRI showing large collection and scapula involvement

**Figure 5:**
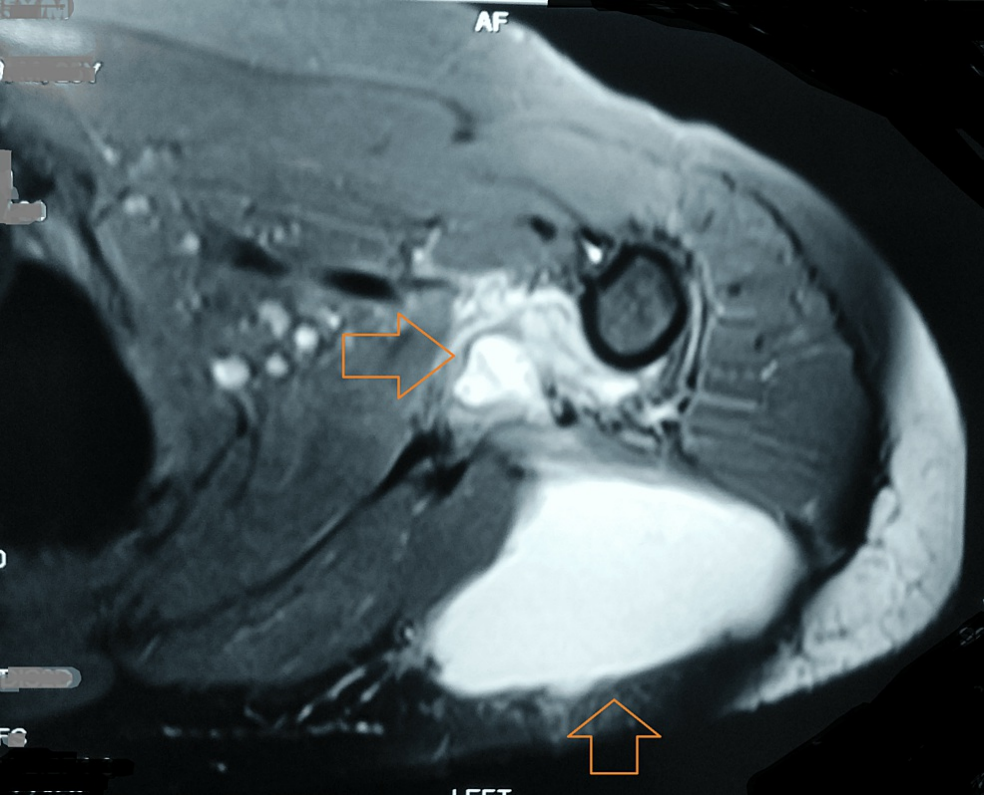
MRI showing left scapula involvement with the formation of a large hyperintense collection located superficially to the left triceps

The magnetic resonance imaging of the left upper arm was suggestive of an area of lysis involving the neck of the scapula, including the inferior cortex. There was evidence of contiguous synovial thickening with abnormal hyperintensity, which extended to involve the anterior and posterior bands of the inferior gleno-humeral ligament in the region of the axillary pouch. There was further extension along the dorsal aspect lateral to the left teres minor that extended up to the subcutaneous plane. There was the formation of a large hyperintense collection located superficially to the left triceps, measuring 5.3 cm x 9 cm x 11.4 cm. There were also small ramifications of the collection extending along the antero-inferior aspect of the left subscapularis. Also, a sub-centimeter-sized level III axillary lymph node was visible. Additionally, an old malunited fracture of the lateral third of the left clavicle was noted (Figures 4-5).

An ultrasound-guided aspiration was done through which approximately 200 ml of pus was removed. A cytological examination of the pus showed necrotic material, caseation, epitheloid cells, and a granulomatous lesion with a few Langhans giant cells. However, the Ziehl-Neelsen stain for acid-fast bacilli was negative. Meanwhile, the cartridge-based nucleic acid amplification of the pus was suggestive of very low levels of *Mycobacterium tuberculosis *with resistance to rifampicin. The presence of *Mycobacterium tuberculosis* was verified by a subsequent, sensitive culture on liquid culture using the Mycobacteria Growth Indicator Tube (MGIT^TM^ 960 system (Becton Dickinson India Pvt. Ltd., Gurugram, India)).

A final diagnosis of rifampicin mono-resistant extrapulmonary tuberculosis of the scapula with cold abscesses was made. After a pre-treatment evaluation, he was initiated on an all-oral longer treatment regimen per the national guidelines (Table 1) [6].

**Table 1:** Antituberculous chemotherapy all-oral longer regimen per his weight OD: once daily

Drug	Route of administration	Dose	Duration
Bedaquiline	Per oral	400 mg X OD followed by 200 mg alternate day	Two weeks and then 22 weeks
Linezolid	Per oral	600 mg X OD	6 months
Levofloxacin	Per oral	1000 mg X OD	24 months
Cycloserine	Per oral	1000 mg X OD	24 months
Clofazimine	Per oral	200 mg X OD	24 months
Pyridoxine	Per oral	100 mg X OD	24 months

For his pain, a tablet of diclofenac was added for once- or twice-daily use. Presently, he is on treatment for two weeks with no major adverse drug reactions to the second-line antituberculous drugs. Moreover, he was regularly counseled for treatment adherence and regular follow-ups. A detailed survey of the family members was also done to rule out tuberculosis, and eligible members were offered a preventive treatment for tuberculosis.

## Discussion

Globally, the prevalence of drug-resistant tuberculosis is rising significantly. There has been an upsurge in the number of rifampicin mono-resistant and multidrug-resistant cases detected in 2022 compared to the previous year [3]. Rifampicin mono-resistant tuberculosis is a type of *Mycobacterium tuberculosis* infection where these bacteria are resistant to only rifampicin [7]. Shoulder involvement is uncommon in skeletal tuberculosis cases (less than 1%); tuberculosis of the scapula constitutes a fraction of this number [8].

Hematogenous or lymphatic dispersion of the bacilli from the main focus of the lung, lymph node, or gut causes bone tuberculosis. In a young patient who is healthy and active, isolated bone involvement without a central focus or history of tuberculosis exposure highlights the question of how the disease was transmitted to such an uncommon location as the scapula [9]. Further, it is a well-known fact that the bacilli can be directly injected into the muscle using a needle after an injection or trauma [10]. Since the scapula is a superficial bone on the dorsal aspect, any sharp object can easily pierce it [9]. The specific fall history in the present case, when he had an accident 1.5 years ago, resulted in abrasion and bruising over the left side's shoulder and upper back region. It's possible that he was infected with the bacteria during this fall, which caused osteomyelitis of the scapula. As demonstrated in this case, sinus development and abscess are typical symptoms of tuberculous osteitis [8].

Most typically, scapular tuberculosis manifests in patients under 30 years of age, occurring equally across genders. Pain and swelling are among the most common symptoms. The most frequent radiological finding is lytic regions surrounded by sclerosis. The most frequently damaged area is the scapular body [5].

Oftentimes, such cases are reported late, and this could be attributed to a lack of constitutional symptoms of tuberculosis, lack of awareness among clinicians and patients, or non-specific findings mimicking several other diseases on the radiographs. Even a magnetic resonance imaging scan can be occasionally misleading [9]. Furthermore, solitary involvement is more common in adults, whereas multifocal involvement is more common in children [11].

Due to non-specific clinical characteristics and incorrect treatment for pyogenic osteomyelitis, the diagnosis is frequently delayed. Multiple myeloma, eosinophilic granuloma, pyogenic or fungal osteomyelitis, and bone metastases are other conditions that can resemble skeletal tuberculosis [12].

Histopathology after tissue biopsy, fine-needle aspiration of the pus, and tissue culture to isolate the *Mycobacterium tuberculosis* or Langhans giant cells, epitheloid granuloma, and caseous necrosis as seen in the present case are ideal for establishing the diagnosis [13]. Moreover, techniques like cartridge-based nucleic acid amplification tests are easily accessible in public and private settings and have a quick turnaround time (two hours), giving results with information about rifampicin resistance [14]. In the present case, cartridge-based nucleic acid amplification tests helped in the diagnosis and prompt treatment initiation.

The management of this condition is mainly conservative with second-line antituberculous drugs [5]. The inclusion of newer drugs like bedaquiline has made the treatment injection-free, which has a direct impact on treatment adherence and outcomes [15]. Nevertheless, surgical intervention can be done in advanced cases for draining the pus, removing the giant sequestra, or when there is a non-satisfactory response to antituberculous drugs at four to six weeks [12].

## Conclusions

A first report of rifampicin-resistant tuberculosis of the scapula with cold abscesses is presented here. Multimodal imaging with a high index of clinical suspicion helped in an early diagnosis, allowing for the initiation of prompt treatment. The case stresses the need for wide reporting of such cases, as even in endemic countries, such cases are scarce. This will also increase awareness among the primary clinicians encountering such cases in primary healthcare settings.
